# Some Comparisons between London and Liverpool

**Published:** 1915-10-02

**Authors:** 


### RESOURCES OF VOLUNTARY HOSPITALS.

### SOME COMPARISONS BETWEEN LONDON AND LIVERPOOL.

In The Hospital of June 12, 1915, page 238, is an article on "The Voluntary Hospitals' Budget." That budget contained a table showing the sources of income from Metropolitan hospitals and dispensaries for ten consecutive years, based upon the Metropolitan Hospital Sunday Fund returns. This exhibition of the income derived from the living and the dead respectively attracted the attention of the Liverpool Council of Voluntary Aid, and we have received a letter from the secretary, Mr. Frederick d'Aeth, who has taken out the figures of fourteen Liverpool hospitals, and has put them into the form of a table based on the same classification as that we published in June last. Mr. d'Aeth points out, as shown in the table, that the proportion of income derived from the living is in Liverpool considerably higher than that in London; that is to say, the percentage the free gifts are of the total income of the Liverpool hospitals is from 16 to 25 greater than it is in London.

In order to make the tables of precise value and accuracy it would be essential for all the figures to be compiled on the same basis, and to include identical items. In the case of the Metropolitan Hospital Sunday Fund figures its statistician would no doubt be prepared to guarantee the published figures in the London return. In the case of Liverpool, however, as Mr. d'Aeth points out, the figures for the years 1907-08 are at any rate incomplete (a fact exhibited by the curiously low proportion of legacies given for the periods in question), due to the figures not having been compiled by the Liverpool Council of Voluntary Aid. Years ago Mr. Chance prepared some most interesting and instructive figures relating to the voluntary subscriptions given to the hospitals of Birmingham, which revealed the small proportion of people who give regularly to the medical charities, compared with the total population. We could wish that every large town had its recognised statistician, who, under the auspices of a council like that of the Liverpool Voluntary Aid, would publish accurately compiled and audited returns of the total voluntary hospital income and expenditure of the town in which he is interested.

The King's Hospital Fund has carried the statistical work of London, so far as the expenditure of the Metropolitan hospitals is concerned, to a point which approaches perfection. The Statistical Returns of that Fund, which is published annually, deals with upwards of one hundred London hospitals. The analyses and tables given are exhaustive, and the information is full of interest and instruction to all who are concerned with the management, maintenance, and cost of voluntary hospitals in this country. It would be interesting to know whether the Liverpool Council of Voluntary Aid has yet published a statistical report of the ordinary expenditure of the fourteen Liverpool hospitals to which Mr. d'Aeth refers in his letter.

